# Variable Virulence of Biotype 3 Vibrio vulnificus due to MARTX Toxin Effector Domain Composition

**DOI:** 10.1128/mSphereDirect.00272-17

**Published:** 2017-07-26

**Authors:** Byoung Sik Kim, Hannah E. Gavin, Karla J. F. Satchell

**Affiliations:** Department of Microbiology-Immunology, Northwestern University Feinberg School of Medicine, Chicago, Illinois, USA; University of Kentucky; University of Valencia; University of South Alabama College of Medicine

**Keywords:** biotype 3, MARTX, Vibrio vulnificus, cytotoxins, mouse, recombination, virulence factors

## Abstract

Vibrio vulnificus is an environmental organism that causes septic human infections characterized by high morbidity and mortality. The annual incidence and global distribution of this pathogen are increasing as ocean waters warm. Clinical strains exhibit variations in the primary virulence toxin, suggesting a potential for the emergence of new strains with altered virulence properties. A clonal outbreak of tilapia-associated wound infections in Israel serves as a natural experiment for the sudden emergence of a new V. vulnificus strain. The effector domain content of the multifunctional autoprocessing RTX (MARTX) toxin of the outbreak-associated biotype 3 (BT3) strains was previously shown to harbor a modification generated by recombination. The modification introduced an actin-induced adenylate cyclase effector domain (ExoY) and an effector domain that disrupts the Golgi organelle (DmX). Here, we report that the exchange of these effector domains for a putative progenitor biotype 1 toxin arrangement produces a toxin that slows the lysis kinetics of targeted epithelial cells but increases cellular rounding phenotypes in response to bacteria. In addition, replacing the biotype 3 toxin variant with the putative progenitor biotype 1 variant renders the resulting strain significantly more virulent in mice. This suggests that the exchange of MARTX effector domains during the emergence of BT3 generated a toxin with reduced toxin potency, resulting in decreased virulence of this outbreak-associated strain. We posit that selection for reduced virulence may serve as a route for this lethal infectious agent to enter the human food chain by allowing it to persist in natural hosts.

**IMPORTANCE** Vibrio vulnificus is a serious infection linked to climate change. The virulence capacity of these bacteria can vary by gene exchange, resulting in new variants of the primary virulence toxin. In this study, we tested whether the emergence of an epidemic strain of V. vulnificus with a novel toxin variant correlated with a change in virulence. We found that restoring the biotype 3 toxin variant to the putative progenitor-type toxin resulted in dramatically increased virulence, revealing that the emergence of the biotype 3 strain could be linked to virulence reduction. This reduced virulence, previously found also in the biotype 1 strain, suggests that reduced virulence may stimulate outbreaks, as strains have greater capacity to enter the human food chain through reduced impact to environmental hosts.

## Abstract (precis)

Vibrio vulnificus is a serious infection linked to climate change. The virulence capacity of these bacteria can vary by gene exchange, resulting in new variants of the primary virulence toxin. In this study, we tested whether the emergence of an epidemic strain of V. vulnificus with a novel toxin variant correlated with a change in virulence. We found that restoring the biotype 3 toxin variant to the putative progenitor-type toxin resulted in dramatically increased virulence, revealing that the emergence of the biotype 3 strain could be linked to virulence reduction. This reduced virulence, previously found also in the biotype 1 strain, suggests that reduced virulence may stimulate outbreaks, as strains have greater capacity to enter the human food chain through reduced impact to environmental hosts.

## INTRODUCTION

The seafood-associated pathogen Vibrio vulnificus causes severe wound and intestinal infections that can progress to tissue necrosis, septicemia, organ failure, and death, often within 48 h of pathogen exposure (1). The pathogen is spreading globally, and more infections are occurring annually as its geographic distribution increases with warming seawaters (2, 3). Biotype 1 (BT1) strains are most commonly associated with clinical infections, while biotype 2 (BT2) strains cause infections in eels (1, 4, 5). From 1996 to 1999, pond-raised tilapia in Israel were linked to an outbreak of wound-associated V. vulnificus infections that was due to a newly emerged clonal variant (6). These biotype 3 (BT3) strains are now in the seas of Israel, where they cause occasional wound infections in fish handlers. The death rate from these infections is 10%, with survivors suffering extreme morbidity, including amputations and long hospitalizations (7).

The primary virulence factor of V. vulnificus BT1, BT2, and BT3 strains is the multifunctional autoprocessing RTX (MARTX) toxin (8–10). These large, secreted, polypeptide toxins have long repeat regions that form pores in eukaryotic cell plasma membranes for the delivery of catalytically active effector domains to cells (11, 12). Among all V. vulnificus isolates, seven different MARTX variants with distinct effector domain repertoires have been identified. In this naturally competent bacterium, the composition and organization of domains is altered by horizontal acquisition of DNA followed by recombination within the *rtxA1* toxin-encoding gene (13). Since the MARTX toxin is a major virulence factor, we surmised that the exchange of effector content in the MARTX toxin, resulting in altered toxin potency, could contribute to the emergence of V. vulnificus strains with outbreak potential (14). The sudden emergence and clonality of BT3 strains serves as a natural experiment for how changes in MARTX effector contents affect disease progression and strain emergence (15). Therefore, in this study, we asked how toxin type impacts virulence during strain emergence by generating a strain in the BT3 background encoding a BT1 C-type toxin from a modified *rtxA1* gene. Specifically, we hypothesized that the acquisition of new effector domains conferred increased virulence on BT3 strains by generating MARTX toxins with increased potency compared to that of the toxin types present in progenitors of BT3. However, our data support the idea that the BT3 toxin type is actually less potent than the C type in this background, suggesting that BT3 emerged in part due to selection for reduced virulence that may enhance persistence in an environmental host.

## RESULTS

### Domain structure of BT3 V. vulnificus and putative progenitor strains.

**FIG 1:**
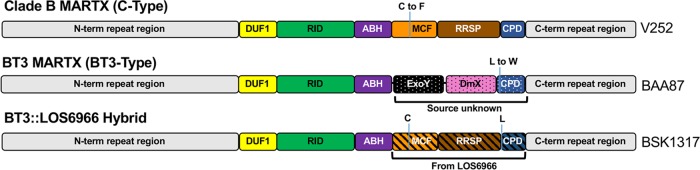
Comparative structures of different V. vulnificus MARTX toxins. Effector arrangements of the BT3-type MARTX toxin in representative strain BAA87 and the C-type toxin found in BT1 strains CMCP6 and LOS6966 and clade B strain V252. Each has five effector domains, as shown by the labels, which are described in the text. The BAA87 hybrid strain modified to have a C-type toxin is also shown. Notable amino acid changes in the clade B and BAA87 toxins are also noted. N-term, N-terminal; C-term, C-terminal.

The 5,208-amino-acid (aa) MARTX toxin in representative BT3 strain BAA87 has five effector domain regions: a domain of unknown function that binds prohibitin (DUF1), a cysteine catalytic protein that inactivates Rho GTPases (RID), an alpha-beta hydrolase enzyme that cleaves phosphatidylinositol-3-phosphate (ABH), an actin-stimulated adenylate cyclase (ExoY), and a cysteine protease that disrupts the Golgi organelle (DmX) (Fig. 1A) (11, 13, 16–18). This effector domain arrangement is unique to BT3 strains. Whole-genome single-nucleotide polymorphism analysis indicates that BT3 strains emerged from a BT1 clade B lineage (19). Analysis for this paper revealed that sequenced representative clade B strain V252 (20) has an *rtxA1* gene that encodes a 5,206-aa MARTX toxin of equal length and 98.8% identity (5,146/5,206 aa) with that of the representative BT1 isolate CMCP6. Unlike the BT3 MARTX toxin, CMCP6 and clade B MARTX toxins have effector domain arrangements of DUF1, RID, ABH, a cysteine protease that induces apoptosis (MCF), and a Ras/Rap1-specific endopeptidase (RRSP) (Fig. 1) (11). This arrangement has been termed the C-type/type I toxin (14, 21).

A notable difference identified between V252 and CMCP6 MARTX toxins is a point mutation at the MCF catalytic cysteine residue of V252. We suggest that the BT3 toxin most likely arose from a C-type toxin as a recombination event that replaced an inactive MCF and neighboring RRSP domains found in the clade B toxin with sequences for the ExoY-DmX domains (Fig. 1). This exchange further incorporated a cysteine protease domain (CPD) with the autoprocessing leucine changed to tryptophan. This likely moves the initial autoprocessing site upstream to the next available Leu; autoprocessing site flexibility has been experimentally demonstrated (22).

### Generation of BT3 strain BAA87 V. vulnificus reverted to carry the putative progenitor C-type MARTX toxin.

We sought to understand the impact of the effector domain exchange by reverting the BT3 toxin back to an unmodified C-type toxin. Among sequenced C-type *rtxA1* genes in our strain collection (14), the sequences flanking the MCF-RRSP-CPD-coding sequence of BT1 strain LOS6966 were most similar to the strain BAA87 *rtxA1* (*rtxA1*_BAA87_) gene sequence, indicating that the LOS6966 *rtxA1* (*rtxA1*_LOS6966_) gene would be a suitable source of DNA for amplification to mediate the genetic exchange (97% nucleotide [nt] identity; 2,416/2,473 nt). The *rtxA1* effector domain region was amplified from LOS6966 chromosomal DNA and cloned into *sacB* counterselection vector pDS132 (23), and the LOS6966-derived gene sequence was recombined into the *rtxA1* gene of BT3 strain BAA87*rif.* The resulting *rtxA1*_BAA87_Ω*rtxA1*_LOS6966_ hybrid strain is referred to as the *rtxA1*_hybrid_ strain hereinafter. In addition, variants of *rtxA1* lacking for ExoY and DmX effector domain DNA sequences (designated *rtxA1*Δ*exoY*, *rtxA1*Δ*dmx*, and *rtxA1*Δ*exoY*Δ*dmx* hereinafter) were generated (Table 1). The Δ*exoY* and Δ*dmx* arrangements were both confirmed to lack relevant toxic activities (see Fig. S1 in the supplemental material; see also reference 17). To focus our studies on *rtxA1*, the gene *vvhA*, encoding a second cytolysin known to impact virulence and lyse cells *in vitro* (8–10), was deleted from all strains; thus, the BAA87 Δ*vvhA* strain is referred to here as the wild type (WT).

10.1128/mSphereDirect.00272-17.1 FIG S1 Strains as indicated were cocultured with CHO cells at an MOI of 20 for 90 min. The concentrations of cyclic AMP (cAMP) in the clarified lysates were determined by enzyme-linked immunosorbent assay (ELISA). Data are pooled from two independent experiments in which assays were performed in triplicate; standard deviations of the results from biological replicates are shown. Results were analyzed by one-way ANOVA followed by multiple comparison test using GraphPad Prism software. *P* values in the figure indicate degree of statistical significance. A portion of these data have been previously published in Ziolo et al. (16). Download FIG S1, PDF file, 0.1 MB. Copyright © 2017 Kim et al. 2017 Kim et al. This content is distributed under the terms of the Creative Commons Attribution 4.0 International license.

**TABLE 1:** Plasmids and bacterial strains used in this study

Strain or plasmid	Relevant characteristic(s)^a^	Reference or source
V. vulnificus strains		
LOS6966 (CDC9342-95)	BT1, clinical isolate, Texas/Louisiana, USA, 1995	A. DePaola, FDA
BAA87*rif*	BT3, human wound isolate, Afula, Israel, 1996, Rif^r^	16
KZ1	BAA87 *rtxA1*::*kan* (Δ*rtxA1*)	16
BS1313	BAA87 Δ*vvhA*	17
BS1314	KZ1 Δ*vvhA*	This study
BS1315	BS1313 *rtxA1*Δ*exoY*	This study
BS1316	BS1313 *rtxA1*Δ*exoY*Δ*dmx*	This study
BS1317	BS1313 *rtxA1*_BAA87_Ω*rtxA1*_LOS6966_ (*rtxA1*_hyrid_)	This study
BS1319	BS1313 *rtxA1*Δ*dmx*	17

E. coli strains		
DH5α	*F*^*−*^ Φ80*lacZ* ΔM15 Δ(*lacZYA-argF*)*U169 recA1 endA1 hsdR17*(r_K_^−^ m_K_^+^) *phoA supE44* *λ*^*−*^ *thi-1gyrA96 relA1*	Laboratory collection
S17-1 *λpir*	*thi pro hsdR hsdM*^+^ *recA*::RP4-2-Tc::Mu-km::Tn*7*; *λpir*, Sm^r^	Laboratory collection

Plasmids		
pDS132	oriR6K *sacB* oriT RP4; Cm^r^	23
pBS1305	*mcf*::*rrsp*::*cpd*_LOS6966_ with flanking regions from BAA87 *rtxA1* in pDS132; Cm^r^	This study
pBS1202	Δ*exoY* in pDS132; Cm^r^	This study
pBS1207	Δ*exoY*Δ*dmx* in pDS132; Cm^r^	This study

a Antibiotic resistance: Ap^r^, ampicillin; Km^r^, kanamycin; Rif^r^, rifampin; Cm^r^; chloramphenicol.

### BAA87 with hybrid toxin shows altered cytopathicity.

**FIG 2:**
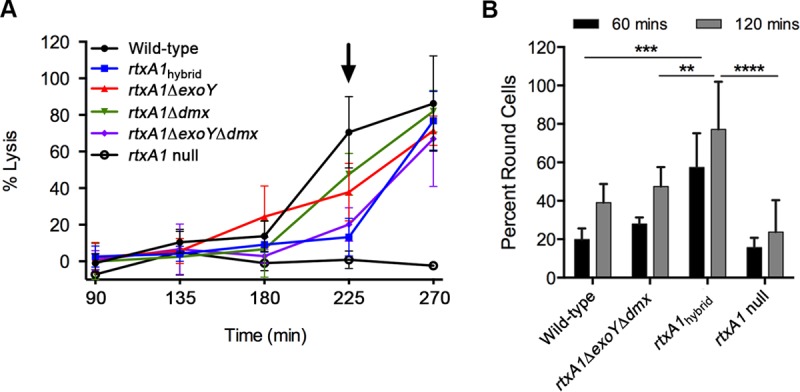
Function of BAA87 with hybrid toxin in cytotoxicity and cytopathicity. (A) HeLa cells were coincubated with V. vulnificus strains as indicated at an MOI of 20. At the indicated times, the LDH release to cell culture supernatant was quantified using the Promega CytoTox96 nonradioactive cytotoxicity assay, and the results are plotted as percent LDH release compared to the results for 100% lysis by 1% Triton X-100. All strains have a deletion in *vvhA*, with BAA87 Δ*vvhA* indicated as the wild type. At *t* = 225 min (arrow), the *rtxA1*_hybrid_, *rtxA1*Δ*exoY*Δ*dmx*, and *rtxA1* null strains induced significantly less lysis than the wild type (*P* < 0.01) as compared by analysis of variance (ANOVA). (B) HeLa cells were coincubated with V. vulnificus at an MOI of 10, and cellular rounding was quantified from digital images using the cell counter plug-in in NIH ImageJ. At both 60 and 120 min, the *rtxA1*_hybrid_ strain induced significantly more rounding than the other strains being compared (**, *P* < 0.05; ***, *P* < 0.01; ****, *P* < 0.001). Data are the mean results ± standard deviations from three experimental sets.

All V. vulnificus strains lyse epithelial cells in culture due to MARTX toxin repeat region pores (12, 24). When incubated with HeLa cells, the *rtxA1*_hybrid_ strain expressed, secreted, and delivered MARTX toxin to target cells, as indicated by the release of lactate dehydrogenase (LDH) (Fig. 2A). The hybrid strain revealed slower cell lysis than the wild-type control (Fig. 2A, arrow at 225 min). Altered kinetics were also observed for *rtxA1*Δ*exoY* and *rtxA1*Δ*dmx* strains, with an additive effect for the *rtxA1*Δ*exoY*Δ*dmx* strain. This suggests that slowed cell lysis by the hybrid is due to the structural or enzymatic changes brought about by loss of ExoY and DmX from the BT3 toxin. In contrast, the *rtxA1*_hybrid_ strain induced more-rapid cell rounding than the WT strain. Interestingly, this indicates increased cell cytopathicity despite delayed lysis. In contrast to the lysis phenotype, the rounding effect is due to the gain of MCF and RRSP, not the loss of ExoY and DmX (Fig. 2B), since accelerated rounding is seen with the hybrid strain but not with the double deletion strain.

### BT3 V. vulnificus with C-type toxin is more virulent than BAA87.

**FIG 3:**
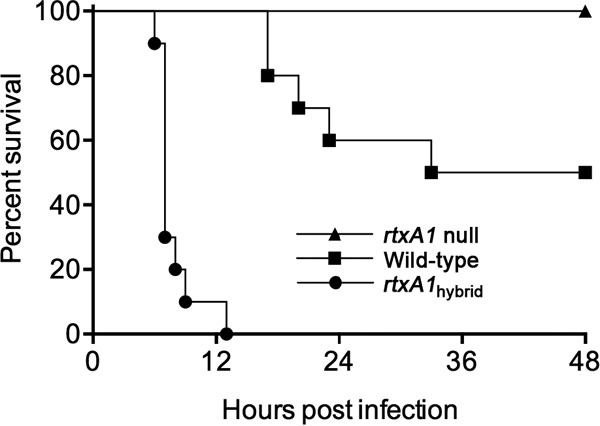
The BT3 BAA87 hybrid with C-type toxin is hypervirulent. Female ICR mice (*n* = 10; pooled data from two experiments conducted with 5 mice per group) were infected s.c. with 5 × 10^6^ CFU of the indicated V. vulnificus strains, and survival was monitored for 48 h. The survival curve of the *rtxA1*_hybrid_-strain-infected group is significantly different from that of the parental wild-type-strain-infected group, as determined by Mantel-Cox log rank test.

Noting that the hybrid toxin conferred delayed lysis kinetics but accelerated rounding kinetics, we tested the effect of the toxin swap on bacterial virulence. The dorsal side of outbred female ICR mice was inoculated by subcutaneous (s.c.) injection with 5 × 10^6^ CFU of bacteria as previously detailed (16). This dose is approximately the 50% lethal dose (LD_50_) for the wild-type strain (BAA87 Δ*vvhA*) (Fig. 2). An isogenic mutant lacking *rtxA1* is significantly attenuated compared to the virulence of the parental strain. One hundred percent of mice survived after *rtxA1* null strain infection. In contrast, all mice inoculated with the *rtxA1*_hybrid_ strain died within 13 h, a significant acceleration compared to the results for the parental strain (Fig. 3).

## DISCUSSION

In this study, we sought to test the hypothesis that naturally occurring effector exchange in MARTX toxins can impact the emergence of epidemic strains. The clonal outbreak of biotype 3 V. vulnificus associated with tilapia fish in Israel provided a natural experiment to test this hypothesis. Contrary to our initial hypothesis, the hybrid strain had increased virulence, indicating that the putative progenitor C-type toxin is actually more potent, in terms of its virulence contribution, than the BT3 toxin of BAA87. This increased virulence occurred despite slower cell lysis kinetics. These results are consistent with recent observations that bacterial virulence outcomes are more likely linked to MARTX effector domain activity than to the lytic cytotoxicity characteristic of MARTX delivery *in vitro* (25). Moreover, these data reveal that a putative BT3 progenitor strain harboring the C-type MARTX toxin could have been more virulent than the current BAA87 if the strains were completely isogenic. Therefore, this experiment suggests that outbreak strains may arise from decreases in the potency of key virulence factors rather than increased potency.

In fact, a reduction of virulence leading to clinical emergence has been previously suggested also for BT1 V. vulnificus strains. In mouse intragastric studies, strains with an RRSP^+^ C-type toxin were 50-fold more virulent than isogenic strains with an RRSP^−^ M-type toxin (14). Loss of the RRSP-coding sequence from *rtxA1* has occurred repeatedly, according to phylogenetic analysis, and RRSP^−^ M-type/type II toxin variants are more common clinically. This occurs despite data showing that RRSP^+^ C-type toxins are more common in oyster isolates (14). To date, RRSP^+^ clade B lineages likewise lack clinical isolates (19). Unfortunately, no retrospective studies in humans have been conducted to correlate strains with disease progression.

Of course, the actual BT1/clade B and BT3 V. vulnificus strains existing today are not isogenic, and this study cannot discount the possibility that other genetic changes may have resulted in altered bacterial virulence potential during the divergence of BT1 and BT3 lineages. Nonetheless, the data certainly reveal that MARTX toxin evolution is not linear in the direction of increased potency, as the BT3 MARTX is notably less potent than the putative progenitor-type toxin tested. Thus, we posit that movement of a strain into human contact may be driven not by hypervirulence but by a reduction of the potency of key virulence factors, including MARTX, that thereby reduces virulence potential in an otherwise highly pathogenic background.

## MATERIALS AND METHODS

### Ethics statement.

This work was performed in strict accordance with the recommendations in the United States Public Health Service regulations and applicable federal and local laws. The methods for modified toxin generation were approved by the Northwestern University Institutional Biosafety Committee. All mouse experiments were conducted in accordance with the protocols approved by the Northwestern University Institutional Animal Care and Use Committee (protocol number IS00000318).

### Reagents, medium, and cell culture.

The bacterial strains and plasmids used in this study are listed in Table 1. Primers used for DNA amplification are listed in Table 2. HeLa cells were obtained directly from the American Type Culture Collection (ATCC) and cultured at 37°C with 5% CO_2_ in Dulbecco’s modified Eagle’s medium (DMEM) supplemented with 10% fetal bovine serum, 50 µg/ml penicillin, and 50 µg/ml streptomycin. Restriction and amplification enzymes and Gibson assembly reagents were purchased from New England Biolabs, and DNA oligonucleotides and gBlocks gene fragments were from Integrated DNA Technologies, Inc.

**TABLE 2:** Primers used in this study

Primer	Sequence (5′ → 3′)
LMPC_1301	CCAAGCGGTGTCTGGCTTATTGCTTGACCGTCCTATG
LMPC_1302	CTCATCCTTGGCAACAACTTCACCTTGCTCGTCCCAG
Los_1305	GCTAGAGAAGATGTACTCTGCGGCAG
Los_1306	CATCTGTTCATTAACAAAGCGTAGGCCG
ExoY_Lic_F	TACTTCCAATCCAATGCGGGGACAAATACAGATGCACCCCAT
ExoY_Lic_R	TTATCCACTTCCAATGCTAATTAATCGCTACCTGCTGGAAATTAGAAAAC
Bio3X_1201	GCTCTATCCAAACGTATTTCAATGTAAATG
Bio3X_1204	CTCCAGTCCACGCATCTTTCTG

### Generation of mutant strains expressing modified MARTX toxin.

All new V. vulnificus strains were generated in a spontaneously rifampin-resistant isolate of BT3 strain BAA87. To generate the hybrid *rtxA1*, a DNA fragment corresponding to the MCF-, RRSP-, and CPD-coding sequence of *rtxA1* was amplified from genomic DNA of LOS6966 using primers LMPC_1301 and LMPC_1302. At the same time, two 500-bp gBlocks gene fragments corresponding to up- and downstream flanking regions of the amplified fragment but containing the sequence matched to the BAA87 *rtxA1* gene were synthesized. These three DNA fragments were assembled into SphI-SacI-digested pDS132 using Gibson assembly master mix, generating plasmid pBS1305. The new DNA arrangement was recombined into BAA87*rif* by conjugation followed by sucrose counterselection as detailed previously (16). The desired mutant was isolated and confirmed by PCR using various primer sets, including LOS_1305 and LOS_1306, ExoY_Lic_F and ExoY_Lic_R, and Bio3X_1201 and Bio3X_1204.

The *rtxA1*Δ*dmx* arrangement was previously described, as listed in Table 1. To generate *rtxA1*Δ*exoY* and *rtxA1*Δ*exoY*Δ*dmx* arrangements, 500-bp gBlocks gene fragment sets corresponding to up- and downstream flanking regions of BAA87 *rtxA1* bp 9703 to 9737 and 9703 to 12180 were assembled into SphI-SacI-digested pDS132 by Gibson assembly, generating plasmids pBS1202 and pBS1207. The new DNA arrangements were recombined into BAA87*rif* by conjugation, followed by sucrose counterselection as detailed previously (17). The desired mutants were isolated and confirmed by PCRs using primer sets ExoY_Lic_F and ExoY_Lic_R and Bio3X_1201 and Bio3X_1204.

All strains were made isogenic with the previously generated strain BS1313 by deletion of *vvhA* to remove this second cytolysin as previously described (17).

### Cytotoxicity assay.

HeLa cells were seeded in a 6-well cell culture dish at a density of 1 × 10^5^ cells/well in complete DMEM (supplemented as described above). On the following day, the cells were washed twice with warmed phosphate-buffered saline (PBS) and the medium replaced with 3 ml of DMEM lacking phenol red, fetal bovine serum (FBS), and antibiotic. Mid-log-phase bacteria were pelleted and resuspended in PBS. One hundred microliters of bacterial suspension was added to each well of HeLa cells to achieve a multiplicity of infection (MOI) of 20. The culture dish containing both HeLa and bacterial cells was centrifuged at 500 × *g* for 3 min and incubated at 37°C with 5% CO_2_. At each time point, 80 µl of cell supernatant was removed from the 3.1-ml total volume per well. Supernatant samples were centrifuged at maximum speed (~15,000 × *g*) for 60 s to pellet any bacteria or cell debris, and 50 µl was subsequently extracted for use in the LDH assay. Experiments were performed in triplicate, with three wells of HeLa cells per bacterial strain. Each experiment also employed (i) three wells of bacterium-free HeLa cells to serve as uninfected controls for spontaneous lysis and (ii) three wells of bacterium-free HeLa cells that were lysed using Triton X-100, as indicated in the CytoTox 96 nonradioactive cytotoxicity assay technical bulletin, to serve as maximum lysis controls. The results for all bacterially infected samples were adjusted for spontaneous lysis and normalized to the percentage of total lysis according to the equations given in the technical bulletin.

### Adenylate cyclase assay.

The assay measuring the increase of adenylate cyclase in V. vulnificus-treated Chinese hamster ovary cells was conducted as previously described (16).

### Mouse wound infection model.

Five-week-old female ICR mice were purchased from Charles River Laboratories, Inc., and allowed to adapt to the new environment for at least 24 h. V. vulnificus strains were grown to mid-log phase (optical density at 600 nm [OD_600_] of approximately 0.5 to 0.8), harvested, and diluted in PBS at 1 × 10^8^ CFU/ml. After slight anesthesia with isoflurane, the mice were subcutaneously injected with 50 µl of bacterial suspension under the dorsal skin. After 6 h postinfection, the mice were monitored at least every 2 h until 24 h and then monitored occasionally after 24 h. Surviving mice without any infection symptoms were euthanized at 96 h postinfection.
