# Supplementary figures and images for: Variable Virulence of Biotype 3 Vibrio vulnificus due to MARTX Toxin Effector Domain Composition
Source: mSphere. 2017 Jul 26;2(4):e00272-17. doi: 10.1128/mSphereDirect.00272-17 (PMC5555677; doi:10.1128/mSphereDirect.00272-17)

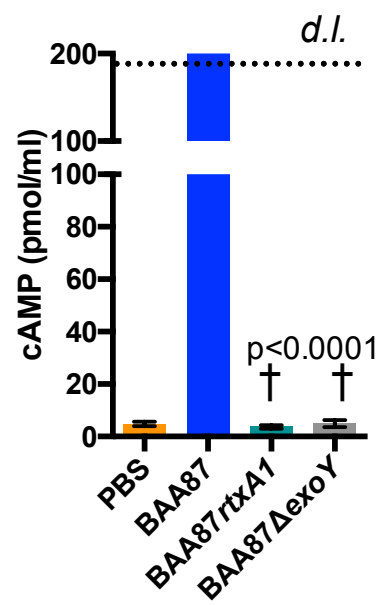

Supplement: FIG S1 [file sph004172327sf1.pdf]
